# Anti-Inflammatory Phenolic Acid Esters from the Roots and Rhizomes of *Notopterygium incisium* and Their Permeability in the Human Caco-2 Monolayer Cell Model

**DOI:** 10.3390/molecules22060935

**Published:** 2017-06-04

**Authors:** Xiu-Wen Wu, Wei Wei, Xiu-Wei Yang, You-Bo Zhang, Wei Xu, Yan-Fang Yang, Guo-Yue Zhong, Hong-Ning Liu, Shi-Lin Yang

**Affiliations:** 1 State Key Laboratory of Natural and Biomimetic Drugs, Department of Natural Medicines, School of Pharmaceutical Sciences, Peking University Health Science Center, Peking University, No. 38, Xueyuan Road, Haidian District, Beijing 100191, China; wuxiuwen0725@126.com (X.-W.W.); gg-993@163.com (W.W.); zybo5288@163.com (Y.-B.Z.); high-xu@163.com (W.X.); yangyanfang@bjmu.edu.cn (Y.-F.Y.); 2 School of Chinese Materia Medica, Jiangxi University of Traditional Chinese Medicine, Nanchang 330004, China; zgy1037@163.com (G.-Y.Z.); lhn0791@139.com (H.-N.L.); slyang3636@126.com (S.-L.Y.); 3 State Key Laboratory of Innovative Drug and Efficient Energy-Saving Pharmaceutical Equipment, Jiangxi University of Traditional Chinese Medicine, Nanchang 330006, China

**Keywords:** *Notopterygium incisum*, phenolic acid esters, 4-methyl-3-*trans*-hexenylferulate, 4-methoxyphenethylferulate, nitric oxide, human intestinal Caco-2 cell, RAW 264.7 macrophage cell

## Abstract

A new ferulic acid ester named 4-methyl-3-*trans*-hexenylferulate (**1**), together with eight known phenolic acid esters (**2**–**9**), was isolated from the methanolic extract of the roots and rhizomes of *Notopterygium incisium*. Their structures were elucidated by extensive spectroscopic techniques, including 2D NMR spectroscopy and mass spectrometry. 4-Methoxyphenethyl ferulate (**8**) NMR data is reported here for the first time. The uptake and transepithelial transport of the isolated compounds **1**–**9** were investigated in the human intestinal Caco-2 cell monolayer model. Compounds **2** and **6** were assigned for the well-absorbed compounds, compound **8** was assigned for the moderately absorbed compound, and compounds **1**, **3**, **4**, **5**, **7**, and **9** were assigned for the poorly absorbed compounds. Moreover, all of the isolated compounds were assayed for the inhibitory effects against nitric oxide (NO) production in the lipopolysaccharide-activated RAW264.7 macrophages model and *L-N*^6^-(1-iminoethyl)-lysine (L-NIL) was used as a positive control. Compounds **1**, **5**, **8**, and **9** exhibited potent inhibitory activity on NO production with the half maximal inhibitory concentration (IC_50_) values of 1.01, 4.63, 2.47, and 2.73 μM, respectively, which were more effective than L-NIL with IC_50_ values of 9.37 μM. These findings not only enriched the types of anti-inflammatory compounds in *N. incisum* but also provided some useful information for predicting their oral bioavailability and their suitability as drug leads or promising anti-inflammatory agents.

## 1. Introduction

*Notopterygii Rhizoma et Radix* (NRR), belonging to Apiaceae, are the dried roots and rhizomes of *Notopterygium incisum* Ting ex H. T. Chang and *N. franchetii* H. de Boiss. and is mainly grown in the Sichuan province of China. NRR is an important ingredient of traditional Chinese medicine recorded in the Pharmacopoeia of People’s Republic of China [1] and has been shown to effectively treat common cold, headache, and rheumatism because of its diaphoretic, analgesic, and anti-inflammatory properties [2,3] for many thousands of years in Asia. Previous studies on the chemical constituents of NRR resulted in the elucidation of multiple components including coumarins, steroids [4,5], polyacetylenes [6], and essential oil [7]. The extent to which these components are effective in the human body depends on their bioavailability and metabolism in vivo. As is well known, intestinal permeability is a crucial factor that influences the bioavailability of drugs, especially of those being administrated orally. Therefore, explaining the intestinal permeability of these components is a critical step towards understanding their potential bioactivity. Human studies have proved that inflammation is critically involved in the disorders of bodies and even contributes to the pathogenesis of several serious diseases such as cancer [8] and neurodegenerative and cardiovascular diseases [9,10]. Hence, the suppression of inflammation in biological systems turned out to be an effective therapeutic strategy and an interesting target in the field of new drug research and development. Nitric oxide (NO) is a short-living free radical that is produced from *L*-arginine by constitutive nitric oxide synthase (cNOS) and inducible nitric oxide synthase (iNOS) within mammalian immune, cardiovascular, and neural systems, where it functions as a signaling or cytotoxic molecule. A low concentration of NO participates in neurotransmission and vasodilation, whereas overproduction of NO by NOS was responsible for inflammation [11]; therefore, NO inhibitor is believed to have therapeutic potential for the treatment of inflammation accompanying overproduction of NO. Our previous studies showed anti-inflammatory activities of some coumarins [12,13], sesquiterpenoids [14], polyacetylenes [15], and neolignans [16,17]. As part of an ongoing effort to search for natural anti-inflammatory agents, herein we describe the isolation and structural elucidation of one new and eight known phenolic acid esters from the dried roots and rhizomes of *N. incisium*, together with predicted absorbability, using a human intestinal Caco-2 cell monolayer model [18] and their inhibitory effects against NO production induced by lipopolysaccharide (LPS) in RAW 264.7 macrophage cells.

## 2. Results and Discussion

### 2.1. Extraction and Isolation

The dried roots and rhizomes of *N. incisium* (2 kg) were powdered and extracted with methanol (MeOH) (6 L × 4 times for 2 h first and 1 h each subsequent time) under reflux. The extracts were combined and then concentrated under reduced pressure to afford a residue (513.0 g, yield 25.7%). The residue was suspended in water and partitioned successively with cyclohexane (CHA), ethyl acetate (EtOAc), and normal butanol (*n*-BuOH) to afford corresponding extracts.

The CHA soluble partition (89.0 g) was subjected to open column chromatography (CC) over a silica gel and eluted successively with CHA–EtOAc (50:1 to 0:1, *v*/*v*) to yield five fractions (Fr.1–5). Fr.1 (3.8 g) was chromatographed on a silica gel column and eluted with petroleum ether (PE)–acetone (50:1, *v*/*v*) to yield four fractions (Fr.1A–1D). Fr.1B (2.1 g) was separated into five subfractions (Fr.1B1–1B5) by reversed-phase semi-preparative HPLC (RP-SP-HPLC) (70% aqueous MeOH, 15 mL/min). By further purification with RP-SP-HPLC, compounds **2** (15.0 mg, 70% aqueous acetonitrile (MeCN), 8 mL/min, *t*_R_ = 26 min, detector UV at 270 nm) and **3** (3.0 mg, 80% aqueous MeOH, 8 mL/min, *t*_R_ = 57 min, detector UV at 270 nm) were obtained from Fr.1B2 and Fr.1B5, respectively. Fr.2 (6.8 g) was fractionated on a silica gel column eluted with PE–EtOAc (15:1, *v*/*v*) to yield three fractions (Fr.2A–2C). Fr.2C (3.5 g) was separated by RP-SP-HPLC (80% aqueous MeOH, 15 mL/min, detector UV at 320 nm) to yield compound **5** (45.0 mg, *t*_R_ = 128 min) and, simultaneously, to obtain subfractions (Fr.2C1–2C5). Fr.2C3 was further purified by RP-SP-HPLC (60% aqueous MeCN, 8 mL/min, detector UV at 270 nm) to yield compounds **6** (4.5 mg, *t*_R_ = 34 min), **1** (5.0 mg, *t*_R_ = 44 min), and **7** (6.0 mg, *t*_R_ = 48 min).

The EtOAc soluble partition (150.6 g) was subjected to CC on silica gel eluted with a gradient of PE–EtOAc (10:1 to 0:1, *v*/*v*) to yield EtOAc fractions 1–4 (EFr.1–4). EFr.2 (1.3 g) was chromatographed on silica gel eluted with chloroform (CHCl_3_)–EtOAc (40:1, *v*/*v*) to yield three fractions (EFr.2A–2C). EFr.2B was separated by RP-SP-HPLC (60% aqueous MeOH, 15 mL/min) to obtain subfractions EFr.2B1–2B6. EFr.2B2 was further purified by RP-SP-HPLC (50% aqueous MeCN, 8 mL/min, detector UV at 320 nm) to yield compounds **4** (5.0 mg, *t*_R_ = 41 min), **8** (4.5 mg, *t*_R_ = 61 min), and **9** (30.0 mg, *t*_R_ = 66 min).

*4-Methyl-3-trans-hexenylferulate* (**1**): White amorphous powder (MeCN–H_2_O); UV (MeOH) λ_max_ (log ε): 321 (3.70) nm (see Supplementary data, Figure S1); IR (KBr) ν_max_: 1729, 1632, 1593, 1454 cm^−1^ (see Supplementary data, Figure S2); ^1^H and ^13^C-NMR data, see Table 1; HR-ESI-MS *m*/*z* 289.1440 [M − H]^−^ (calcd. for C_17_H_21_O_4_, 289.1440).

*4-Methoxyphenethylferulate* (**8**): Pale yellow powder (MeCN–H_2_O); UV (MeOH) λ_max_ (log ε): 214 (3.68) nm (see Supplementary data, Figure S10); IR (KBr) ν_max_: 1705, 1632, 1600, 1464 cm^−1^ (see Supplementary data, Figure S11); ^1^H and ^13^C-NMR data, see Table 1; ESI-MS *m*/*z* 327 [M − H]^−^.

### 2.2. Structural Elucidation of Isolated Compounds ***1***–***9***

**Figure 1:**
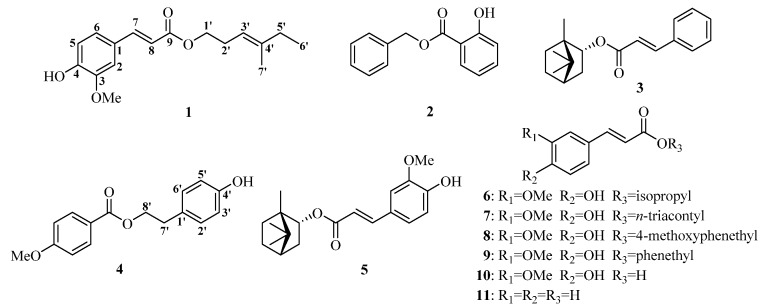
The structures of compounds **1**–**9** isolated from the roots and rhizomes of *N. incisium* and two related compounds **10** and **11**.

The CHA and EtOAc extracts were subjected to column chromatography repeatedly to yield nine phenolic acid esters, which included a new one, 4-methyl-3-*trans*-hexenylferulate (**1**), together with eight known ones, benzylsalicylate (**2**) [19], bornylcinnamate (**3**) [20], *p*-hydroxyphenethyl anisate (**4**) [4], (−)-bornylferulate (**5**) [21], isopropylferulate (**6**) [22,23], *trans*-triacontyl-4-hydroxy-3-methoxycinnamate (**7**) [24], 4-methoxyphenethylferulate (**8**) [25,26], and phenethylferulate (**9**) [27]. Their chemical structures are shown in Figure 1. This is also the first report on the isolation of compounds **2**, **3**, **7**, and **8** from *N. incisium* and NMR data of **8**.

**Figure 2:**
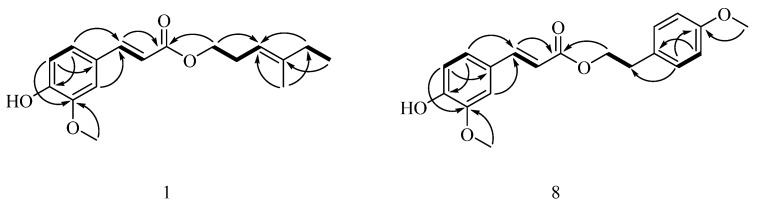
^1^H–^1^H COSY (−) and key HMBC (→; from H to C) correlations of compounds **1** and **8**.

Compound **1** was obtained as a white amorphous powder. Its negative HR-ESI–MS showed the [M − H]^−^ ion at *m*/*z* 289.1440 (see Supplementary data, Figure S3), which corresponded to a molecular formula of C_17_H_22_O_4_, indicating 7 degrees of unsaturation. The IR spectrum showed maximum absorption bands due to a hydroxyl group (3420 cm^−1^), an ester carbonyl bond (1729 cm^−1^), a double bond (1632 cm^−1^), and an aromatic ring (1593, 1514 cm^−1^). The low field of its ^1^H-NMR spectrum (see Supplementary data, Figure S4) showed characteristic signals (Table 1) of a 1,3,4-trisubstituted benzene moiety (ABX spin system) at δ_H_ 6.92 (1H, d, *J* = 8.1 Hz, H-5), 7.07 (1H, dd, *J* = 8.2, 1.7 Hz, H-6), and 7.03 (1H, d, *J* = 1.7 Hz, H-2), and one trans-double bond signals at δ_H_ 7.61 (1H, d, *J* = 15.9 Hz, H-7) and 6.29 (1H, d, *J* = 15.9 Hz, H-8). In its ^13^C-NMR spectrum (see Supplementary data, Figure S5), one carbon signal at δ_C_ 167.3 (C-9) assignable to one ester group was observed. The above-mentioned information suggested the presence of one ferulic acid moiety in **1**, which indicated that **1** was a ferulic acid derivative. With the aid of its DEPT and 2D NMR spectra (see Supplementary data, Figures S6–S9), other NMR resonances assignable to two methylene at δ_H_ 2.41 (2H, q, *J* = 6.9 Hz, H-2′) and δ_C_ (27.6, C-2′), as well as δ_H_ 2.02 (2H, q, *J* = 7.5 Hz, H-5′) and δ_C_ (32.4, C-5′); one oxygen-bearing methylene at δ_H_ 4.17 (2H, t, *J* = 7.1 Hz, H-1′) and δ_C_ (64.2, C-1′); one olefinic methine at δ_H_ 5.16 (1H, t, *J* = 7.6 Hz, H-3′) and δ_C_ (117.7, C-3′); one olefinic methyl group at δ_H_ 1.65 (3H, s, H-7′) and δ_C_ (16.1, C-7′); and one methyl group at δ_H_ 1.00 (3H, t, *J* = 7.5 Hz, H-6′) and δ_C_ (12.7, C-6′) were confirmed. The connection to each other of these functional groups was established by analysis of the ^1^H–^1^H COSY spectrum and the key HMBC correlations (Figure 2). Thus, the spectroscopic evidence was consistent with the assignment of the structure of **1** as (2*E*)-3-(4-hydroxy-3-methoxyphenyl)-2-propenoic acid (3*E*)-4-methylhex-3-enyl ester (Figure 1) and **1** was given the trivial name 4-methyl-3-*trans*-hexenylferulate.

Although 4-methoxyphenethylferulate (**8**) derived from combinatorial enzymatic synthesis catalyzed by *Candida antarctica* lipase B [25,26] is a known compound, NMR data has not been reported thus far. Thorough exhaustive interpretation of the ^1^H and ^13^C-NMR, DEPT, ^1^H–^1^H COSY, HSQC, and HMBC spectra (see Supplementary data, Figures S12–S17), the complete unambiguous assignments for all the ^1^H and ^13^C-NMR signals (Table 1) of **8** were performed for the first time.

### 2.3. Transport of Phenolic Acid Esters ***1***–***9*** in the Human Intestinal Caco-2 Cell Monolayer Model

Intestinal permeability of phenolic acid esters **1**–**9** was evaluated by using human intestinal Caco-2 cell monolayer model [18]. The HPLC analytical methods for the phenolic acid esters had been validated (see Table S1). The bilateral (apical side (AP) → basolateral side (BL) and BL → AP) apparent permeability coefficients (P_app_) values of compounds **1**–**9** are summarized in Table 2. The *P*_app AP→BL_ values of compounds **2** and **6** in the present study were well over 10^−5^ cm/s, which were comparable to that of propranolol (2.29 × 10^−5^ cm/s), a well-transported marker of the transcellular pathway [18], indicating their good absorption, whereas the *P*_app AP__→BL_ magnitudes of compounds **1**, **4**, and **9** were below 10^−6^ cm/s, which were comparable to that of atenolol (5.54 × 10^−7^ cm/s), a poor-transported marker of the paracellular pathway [18], so compounds **1**, **4**, and **9** were assigned for the poorly absorbed compounds.

**Figure 3:**
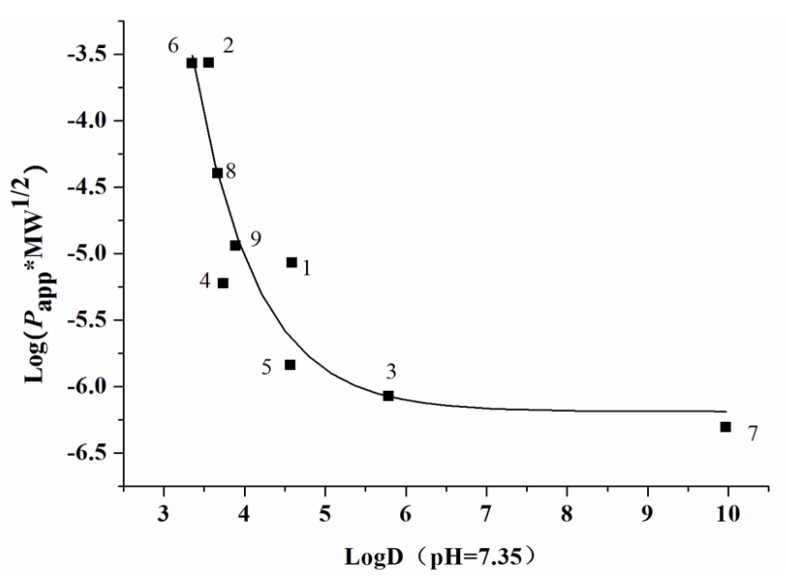
The relationship between log (*P*_app AP__→BL_ × MW^1/2^) and log D (pH = 7.35) for nine phenolic acid esters (**1**–**9**).

The ***P*_app AP_**_→**BL**_ magnitude of compound **8** was a quantitative degree of 10^−6^ cm/s, which fell in between propranolol and atenolol; compound **8** was thereby assigned for the moderately absorbed compound. Compounds **3**, **5**, and **7** were found to hardly permeate Caco-2 monolayers with *P*_app AP→BL_ magnitudes <10^−7^ cm/s. The efflux ratios of the above phenolic acid esters except compounds **3**, **4**, and **7** were within the range of 0.8–1.5. Physicochemical characters such as log D (logarithm of octanol–water partition coefficient) and MW (molecular weight) are generally utilized for the prediction of the permeability of compounds [28]. The log D values at pH 7.35 of nine phenolic acid esters, calculated with Pallas 3.3.2.6 ADME/Tox Software (CompuDrug, Bal Harbor, FL, USA), as well as their MW values are shown in Table 2. Herein, an inverse sigmoid trendline of log (*P*_app AP→BL_ × MW^1/2^) versus logD was plotted (Figure 3) with Origin Pro 7.5 SR1 (Origin Lab Corporation, Northampton, MA, USA) to elucidate the structure–permeability relationship of these phenolic acid esters. The permeability of phenolic acid esters presented a downward trend as log D values (>3) increased, indicating that a lipophilicity that is too high may result in low membrane permeability.

### 2.4. Inhibitory Activity of Compounds ***1***–***9*** on NO Production

As part of our project to find natural structures with inhibitory activity on overproduction of NO, all of the isolated compounds were evaluated against NO release in LPS-activated RAW264.7 macrophage cell model [12,14]. The compounds **1**–**9** were initially assayed for cytotoxic effects in RAW264.7 cells by the 3-(4,5-dimethyl-2-thiazolyl)-2,5-diphenyl-2*H*-tetrazolium bromide (MTT) assay. The cell viability less than 95% of control was considered toxic. The results demonstrated that compounds **1**–**6**, **8**, and **9** showed no toxicity at any tested concentrations (0.78125–25 μM for compounds **1** and **5**, 3.125–100 μM for compounds **2**, **3**, and **4**, and 1.5625–50 μM for compounds **6**, **8**, and **9**), except compound **7**, which had cytotoxicity at concentrations from 0.25 to 8 μM. As is shown in Table 3, compounds **1**, **4**, **5**, **6**, **8**, and **9** showed potent inhibition with the half maximal inhibitory concentration (IC_50_) values of 1.01, 11.11, 4.63, 12.62, 2.47, and 2.73 μM, respectively, comparable to the positive control *L*-*N*^6^-(1-iminoethyl)-lysine (L-NIL) with an IC_50_ value of 9.37 μM. Compounds **2** and **3** showed moderate activity with IC_50_ values of 53.69 and 70.50 μM, respectively, which were inferior to L-NIL. The effect on NO production of compound **7** was not further evaluated for its damage on cells at all test concentrations. Notably, NO inhibition activities of compounds **1**, **5**, **8**, and **9** were more significant (*p* < 0.001, *p* < 0.05, *p* < 0.001, *p* < 0.001) than that of L-NIL. We also evaluated two related compounds, ferulic acid (**10**, Figure 1) and cinnamic acid (**11**, Figure 1), to discuss the initial structure–activity relationship. Ferulic acid, with an IC_50_ value of 67.94 μM, exhibited higher inhibitory activity than that of cinnamic acid (IC_50_ > 200 μM). Comparing compound **5** with compound **3** in structures and IC_50_ values, it was found that the replacement of cinnamoyl group with feruloyl group significantly increased by an order of magnitude in the activity, suggesting a more significant effect of the feruloyl group than the cinnamoyl group on the inhibitory activity. Compared to compounds **2** and **4**, compound **9** more effectively inhibited NO production, suggesting that the feruloyl group plays a more important role than do the salicyloyl or anisoyl groups in exerting the activity. The above-mentioned information confirmed that the feruloyl group was critical for maintaining or enhancing NO inhibition activities. In addition, all ferulic acid esters that were tested (compounds **1**, **5**, **6**, **8**, and **9**) exhibited stronger NO inhibition than ferulic acid (**10**), and the activity of cinnamic acid ester (**3**) was also superior to that of cinnamic acid (**11**), suggesting that the ester moiety was a requirement in phenolic acid derivatives for the activity. For the five ester of ferulic acid, the increasing order of the inhibition activity against NO production is as follows: **6** < **5** (or **8**, **9**)< **1**. Comparing the activity of compounds **1** and **6**, which possess hydrocarbyl substitution but differ in the length of carbon chain, it was obvious that the long hydrocarbon chain was responsible for the enhanced activity. The data of compounds **8** and **9** suggested that methoxyl substitution of phenethyl moiety had little influence on the activity.

Considering the good activity of compounds **1**–**6**, **8**, and **9**, especially compounds **1**, **5**, **8**, and **9**, which were superior to L-NIL in NO production inhibition (Table 3), these phenolic acid esters can become leading candidates to research and development agents for the treatment of inflammatory disease accompanying overproduction of NO. However, given the absorption properties, only compounds **2**, **6**, and **8** are considered to have therapeutic potential for the treatment of inflammation accompanying overproduction of NO due to their good or moderate membrane permeability in Caco-2 cell model (Table 2). Both ferulic acid ester derivatives, 1-*O*-feruloyl-2-*O*-*p*-coumaroylglycerol and 1,3-*O*-diferuloylglycerol, significantly decreased the production of NO in LPS-stimulated mouse macrophage RAW264.7 cells in a dose-dependent manner with IC_50_ values of 9.12 ± 0.72 and 12.01 ± 1.07 µM, respectively, through acting on the NF-κB/MAPKs pathway [29], which provides guidance on further research on the underlying mechanism of these phenolic acid esters from *N. incisium* exerting NO inhibition.

## 3. Experimental Section

### 3.1. Plant Material

The roots and rhizomes of *Notopterygium incisium* were gathered from the Danba county of the Sichuan province of China and were identified by Dr. Shun-Yuan Jiang of Sichuan Academy of Chinese Medicine Sciences (Chengdu, China). A voucher specimen (No. QH201409) was deposited in State Key Laboratory of Natural and Biomimetic Drugs (Peking University, Beijing, China).

### 3.2. Chemicals and Reagents

HPLC grade MeOH and MeCN were purchased from Fisher Scientific (Fair lawn, NJ, USA). Analytical grade MeOH, PE, CHA, EtOAc, CHCl_3_, *n*-BuOH, acetone, and Hank’s Balanced Salts Solution (HBSS) were purchased from Beijing Chemical Works (Beijing, China). Water was purified by a Mili-system (Millipore, Bedford, MA, USA). Propranolol, atenolol, dimethyl sulfoxide (DMSO), LPS, Griess reagent, NaNO_2_, L-NIL, and MTT were purchased from Sigma-Aldrich Co. (St Louis, MO, USA). Penicillin-streptomycin solution was obtained from Suolaibao Technology Ltd. (Beijing, China). Dulbecco’s Modified Eagle’s Medium (DMEM), fetal bovine serum (FBS), phosphate buffered saline (PBS), nonessential amino acids (NEAA), and trypsin were supplied by Gibco^®^ Laboratories (Life Technologies Inc., Grand Island, NY, USA). Ferulic acid and cinnamic acid were supplied by Natural Product Sample Library in State Key Laboratory of Natural and Biomimetic Drugs of Peking University (Beijing, China).

### 3.3. Instrumental Analyses

Open column chromatography (CC) separation was carried out using silica gel (200–300 mesh; Qingdao Marine Chemical Co., Qingdao, China). Thin layer chromatography was conducted on silica gel GF_254_ plates (Merck, Darmstadt, Germany). Isolation of compounds was performed on an RP-SP-HPLC, which was conducted on a Beijing CXTH 3000 system (Beijing Chuang Xin Tong Heng Sci. Technol. Co. Ltd., Beijing, China) with two P3050 pumps, UV3000 ultraviolet-visible detector, A1359 liquid handler with a loop of 5 mL. The LC Workstation was CXTH 3000 Chromsoftware. A preparative Phenomenex Prodigy C_18_ column (250 × 21.2 mm i.d., 10 μm; Phenomenex, Torrance, CA, USA) equipped with a C_18_ guard column (8 × 4 mm i.d., 5 μm; Dikma, China) was used for isolation and purification of the compounds. ^1^H (400 MHz), ^13^C (100 MHz) and 2D NMR spectra were run on a Bruker AV 400 spectrometer (Bruker, Karlsruheb, Baden-Wuerttemberg, Germany) with tetramethylsilane as an internal standard and CDCl_3_ as solvent. Electron spray ionization mass spectrometry (ESI-MS) data were obtained using a triple quadrupole mass spectrometer 8050 system (Shimadzu Corp., Kyoto, Japan). High-resolution electron spray ionization mass spectrometry (HR-ESI-MS) data were obtained using a Waters Xevo G2 Q-TOF mass spectrometer (Waters, Milford, MA, USA). IR spectra were recorded on a Nexus 470 FT-IR spectrometer (Thermo Nicolet, Inc., Madison, WI, USA) with KBr disks. UV spectra were acquired on a Cary 300 UV-vis spectrophotometer (Varian, Inc., Palo Alto, CA, USA) in MeOH solution.

Quantitative analysis of nine phenolic acid esters was performed on a Dionex Ultimate™ 3000 UHPLC system (Dionex Corp., Sunnyvale, CA, USA), comprised of Ultimate 3000 pump, autosampler, column compartment, and diode array detector. The signals were acquired and processed applying a Chromeleon version 6.80 software (Dionex Corp., Sunnyvale, CA, USA). HPLC separation was performed on a C_18_ Luna^®^ column (250 mm × 4.60 mm i.d., 5 μm) (Phenomenex, Torrance, CA, USA). The mobile phase consisted of MeOH and water (*v*/*v*) in 90:10 for compounds **3**, **5**, and **7**, 80:20 for compounds **1**, **2**, **8**, and **9**, and 70:30 for compounds **4** and **6**, with a flow rate of 1 mL/min. The detection wavelength was set at 305 nm for **2**, 270 nm for **3**, 259 nm for **4**, 325 nm for **1** and **5**–**9**. Column oven was maintained at 25 °C, and the injected volume was 20 μL. The calibration curves were constructed by plotting peak area (*y*, mAU × min) versus concentration (*x*, µM). The linear equations across 1–75 µM were *y* = 0.0087*x* − 0.0041 with *r*^2^ of 0.9994 for **1**, *y* = 0.0066*x* − 0.0013 with *r*^2^ of 0.9999 for **2**, *y* = 0.0667*x* − 0.0269 with *r*^2^ of 0.9997 for **4**, *y* = 0.0917*x* + 0.0040 with *r*^2^ of 0.9998 for **5**, *y* = 0.0614*x* − 0.0038 with *r*^2^ of 0.9999 for **6**, *y* = 0.0623*x* + 0.0051 with *r*^2^ of 0.9999 for **8**, and *y* = 0.0639*x* − 0.0464 with *r*^2^ of 0.9978 for **9**. The amounts of **3** and **7** in the receiving chambers were below the lower limit of detection (LLOD). Quantification was carried out by peak area measurements in comparison with the calibration curves. Methodology was examined for precision, accuracy, recovery, and stability (see Supplementary data, Table S1) and was demonstrated to meet the requirements of determination.

### 3.4. Cell Culture

The human intestinal Caco-2 cell line (ATCC #HTB-37) was purchased from American Type Culture Collection (ATCC, Rockville, MD,, USA). The murine macrophage cell line RAW264.7 cell (3111C0001CCC000146) was obtained from the Cell Resource Center, IBMS, CAMS/PUMC (Beijing, China). The cell culture was carried out in a Sanyo MCO-15 AC carbon dioxide (CO_2_) incubator (Sanyo Electric Co., Ltd., Osaka, Japan). The integrity of the Caco-2 cell monolayer was examined by measuring the transepithelial electrical resistance (TEER) with an epithelial voltohmmeter (EVOM, World Precision Instrument, Sarasota, FL, USA) [18].

### 3.5. Caco-2 Cell Permeability

The Caco-2 cells were maintained in DMEM containing 10% FBS, 1% NEAA (100×), 100 units/mL of penicillin, and 100 µg/mL of streptomycin, in a constant humidity atmosphere of 5% CO_2_ and 95% air at 37 °C. For confluence and differentiation, cells were seeded at a density of 1 × 10^5^ cells/cm^2^ into 12-well Transwell plates (insert diameter 12 mm, pore size 3.0 μm, membrane growth area 1.12 cm^2^, Costar^®^ #3402) and were allowed to grow for 21 days before the permeation experiment. On Day 21, the monolayers with TEER values >500 Ω·cm^2^ were qualified for the transport experiment. The transport study was initiated by the careful removal of the culture medium from AP and BL side of the inserts. Caco-2 monolayers were rinsed twice with pre-warmed HBSS and were incubated by pre-warmed HBSS for 30 min at 37 °C. Stock solutions of test phenolic acid esters were prepared in DMSO and diluted to 50 µM with HBSS. The final DMSO concentration was less than 2%, a concentration that did not alter cell viability or permeability. The assayed solutions (50 µM) were added to the AP side (0.5 mL, for absorption transport) or BL side (1.5 mL, for efflux transport) of the inserts, while the receiving chamber contained the corresponding volume of HBSS. Incubation was performed at 37 °C for 90 min, with shaking at 50 rpm. Samples were collected from the inserts, then frozen, lyophilized, redissolved in MeOH, and injected into HPLC system for quantitative analysis.

The *P*_app_ values were calculated as the following equation:

*P*_app_ = (dQ/dt) × (1/A) × (1/C_0_) (cm/s) (1)

where dQ/dt is the rate of the appearance of the test compound on the receiver compartment (μmol/s), C_0_ is the initial test compound concentration on the donor compartment (μmol/mL), and A is the surface area of Caco-2 monolayer (cm^2^).

### 3.6. NO Inhibitory Assay

The NO inhibitory assay was carried out according to the previous method [12,14]. Briefly, RAW 264.7 cells were maintained in DMEM containing 10% FBS, in a constant humidity atmosphere of 5% CO_2_ and 95% air at 37 °C, seeded in 96-well culture plates (Costar^®^ #3599, Cambridge, MA, USA) at a density of 3 × 10^4^ cells/well for 12 h, and then stimulated with LPS (1 μg/mL) and treated with various concentrations of assayed compounds for 24 h. After that, the cell culture supernatant (100 μL) was collected to react with Griess reagent (100 μL) for 15 min at room temperature. The nitrite in culture medium was measured as an indicator of NO production. NaNO_2_ was used to generate a standard curve, and NO production was determined by measuring the optical density at 540 nm in comparison with the standard curve. Thermo Multiskan MK 3 Automated Microplate Reader was Thermo-Labsystems (Franklin, MA, USA). The experiments were performed in parallel three times, and L-NIL was used as a positive control. Cell viability (>95%) was assessed by using an MTT assay. The IC_50_ values were calculated by the software SPSS 16.0 (SPSS Inc., Chicago, IL, USA).

### 3.7. Statistical Analysis

The results presented in this study were the averages of at least three replicates and were presented as means ± SD. The data were analyzed by either *t*-test or nonparametric test after analysis of variance using SPSS 16.0. The level of significance was set at *p* < 0.05.

## 4. Conclusions

In conclusion, a new ferulic acid ester (**1**) along with eight known phenolic acid esters (**2**–**9**) was isolated from the roots and rhizomes of *N. incisium*. The absorption properties of these phenolic acid esters were estimated with human intestinal Caco-2 cell monolayer model. Moreover, these phenolic acid esters except for compound **7** exhibited significantly inhibitory effects on NO production and particularly ferulic acid esters may be considered as potential therapeutic agents in inflammatory diseases associated with NO overproduction. Considering the beneficial effects of compounds **2**, **6**, and **8** on the inhibition of NO production and their possibility of crossing the intestinal barrier, compounds **2**, **6**, and **8** may be considered to contribute to the anti-inflammatory activity of the roots and rhizomes of *N. incisum*.

## Figures and Tables

**Table 1:** ^1^H (400 MHz) and ^13^C (100 MHz)-NMR data for compounds **1** and **8** in CDCl_3_.

No.	1		No.	8	
	*δ*_H_ (*J* in Hz)	*δ*_C_ (mult.) ^a^		*δ*_H_ (*J* in Hz)	*δ*_C_ (mult.) ^a^
1	–	127.1, C	1	–	127.1, C
2	7.03, d (1.7)	109.3, CH	2	7.02, d (2.1)	109.4, CH
3	–	146.7, C	3	–	146.8, C
4	–	147.9, C	4	–	148.0, C
5	6.92, d (8.1)	114.7, CH	5	6.92, d, 8.2	114.8, CH
6	7.07, dd (8.1, 1.7)	123.0, CH	6	7.07, dd (8.2, 2.1)	123.1, CH
7	7.61, d (15.9)	144.7, CH	7	7.60, d (15.9)	144.9, CH
8	6.29, d, (15.9)	115.7, CH	8	6.27, d (15.9)	115.6, CH
9	–	167.3, C	9	–	167.2, C
3-OCH_3_	3.93, s	55.9, CH_3_	3-OCH_3_	3.93, s	56.0, CH_3_
4-OH	5.84, s	–	4-OH	5.86, s	–
1′	4.17, t (7.1)	64.2, CH_2_	1′	–	130.0, C
2′	2.41, q (6.9)	27.6, CH_2_	2′, 6′	7.18, d (8.8)	129.9, CH
3′	5.16, t (7.6)	117.7, CH	3′, 5′	6.86, d (8.8)	114.0, CH
4′	–	140.2, C	4′	–	158.4, C
5′	2.02, q (7.5)	32.4, CH_2_	7′	2.96, t (7.0)	34.4, CH_2_
6′	1.00, t (7.5)	12.7, CH_3_	8′	4.38, t (7.0)	65.2, CH_2_
7′	1.65, s	16.1, CH_3_	4′-OCH_3_	3.79, s	55.3, CH_3_

^a^ Attached protons determined by DEPT experiment.

**Table 2:** The bidirectional *P*_app_ values of compounds **1**–**9** in Caco-2 cell monolayer (*n* = 4) ^a^.

No.	*P*_app AP__→BL_^b^ (×10^−6^ cm/s)	*P*_app BL__→__AP_^c^ (×10^−6^ cm/s)	Efflux Ratio ^d^	MW	Log D (pH = 7.35)
**1**	0.50 ± 0.12	0.62 ± 0.12	1.24	290	4.59
**2**	18.04 ± 1.63	13.45 ± 0.63	0.75	228	3.56
**3**	<0.05	<0.02	–	284	5.79
**4**	0.36 ± 0.04	0.05 ± 0.01	0.15	272	3.74
**5**	0.08 ± 0.02	0.13 ± 0.02	1.69	330	4.57
**6**	17.59 ± 2.27	13.85 ± 2.74	0.79	236	3.35
**7**	<0.02	<0.01	–	614	9.97
**8**	2.21 ± 0.07	2.55 ± 0.44	1.15	328	3.67
**9**	0.66 ± 0.16	0.70 ± 0.18	1.06	298	3.89

^a^ The incubation time was up to 90 min. ^b^ Transport of test compounds from AP to BL direction. ^c^ Transport of test compounds from BL to AP direction. ^d^ The ratio of *P*_app BL__→AP_ to *P*_app AP__→BL_.

**Table 3:** Inhibition of compounds **1**–**9** on NO production (*n* = 3).

No.	IC_50_ (μM)	No.	IC_50_ (μM)	No.	IC_50_ (μM)
**1**	1.01 ± 0.08 ***	**5**	4.63 ± 1.73 *	**10**	67.94 ± 0.91
**2**	53.69 ± 4.13	**6**	12.62 ± 2.80	**11**	>200
**3**	70.50 ± 25.86	**8**	2.47 ± 0.64 ***	L-NIL	9.37 ± 1.57
**4**	11.11 ± 1.43	**9**	2.73 ± 0.58 ***		

L-NIL: *L*-*N*^6^-(1-iminoethyl)-lysine; * *p* < 0.05 vs. L-NIL; *** *p* < 0.001 vs. L-NIL.

## Supplementary Materials

Supplementary materials are available online: Figures S1–S17 and Table S1.
