# Supplementary material for: Anti-Inflammatory Phenolic Acid Esters from the Roots and Rhizomes of Notopterygium incisium and Their Permeability in the Human Caco-2 Monolayer Cell Model
Source: Molecules. 2017 Jun 4;22(6):935. doi: 10.3390/molecules22060935 (PMC6152638; doi:10.3390/molecules22060935)
Supplement: Supplementary file 1 [file molecules-22-00935-s001.pdf]

# Supplementary Material:

# Anti-Inflammatory Phenolic Acid Esters from the Roots and Rhizomes of *Notopterygium incisium* and Their Permeability in the Human Caco-2 Monolayer Cell Model

**Xiu-Wen Wu, Wei Wei, Xiu-Wei Yang*, You-Bo Zhang, Wei Xu, Yan-Fang Yang, Guo-Yue Zhong, Hong-Ning Liu, Shi-Lin Yang**

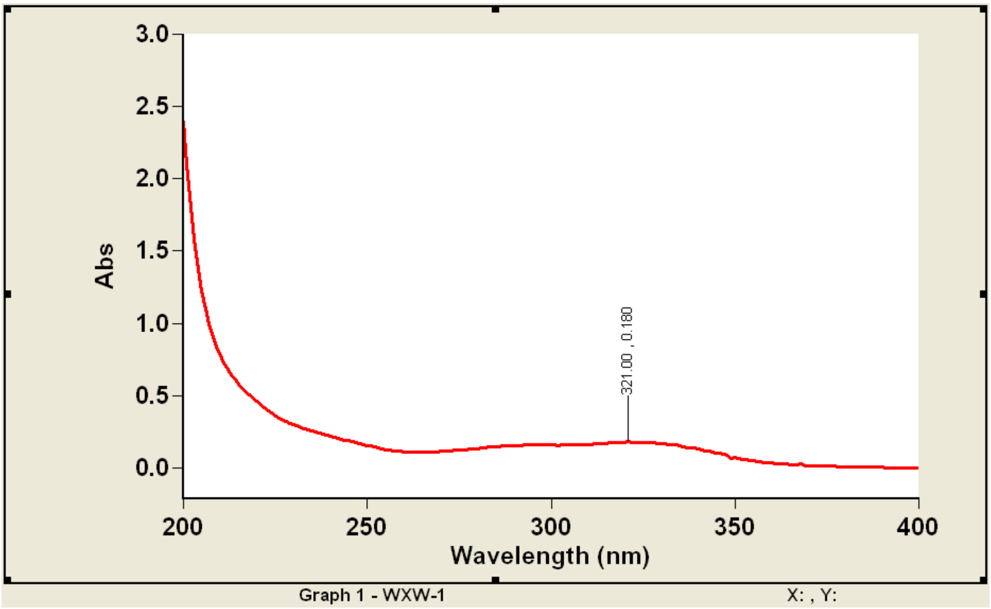


**Figure S1**. UV spectrum of **1** in MeOH.

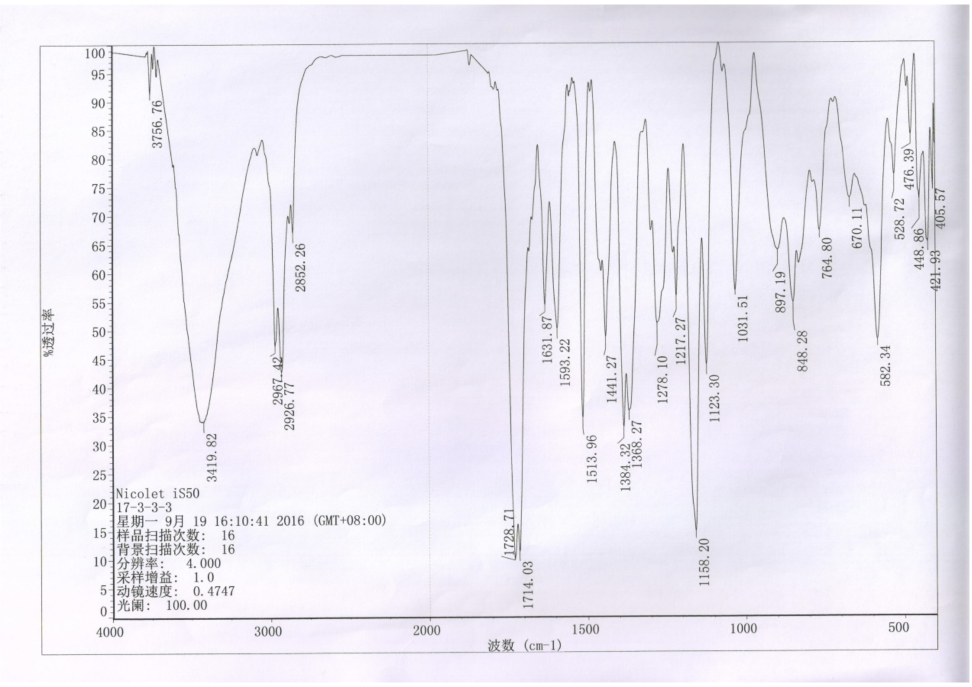


**Figure S2**. IR spectrum of **1**.

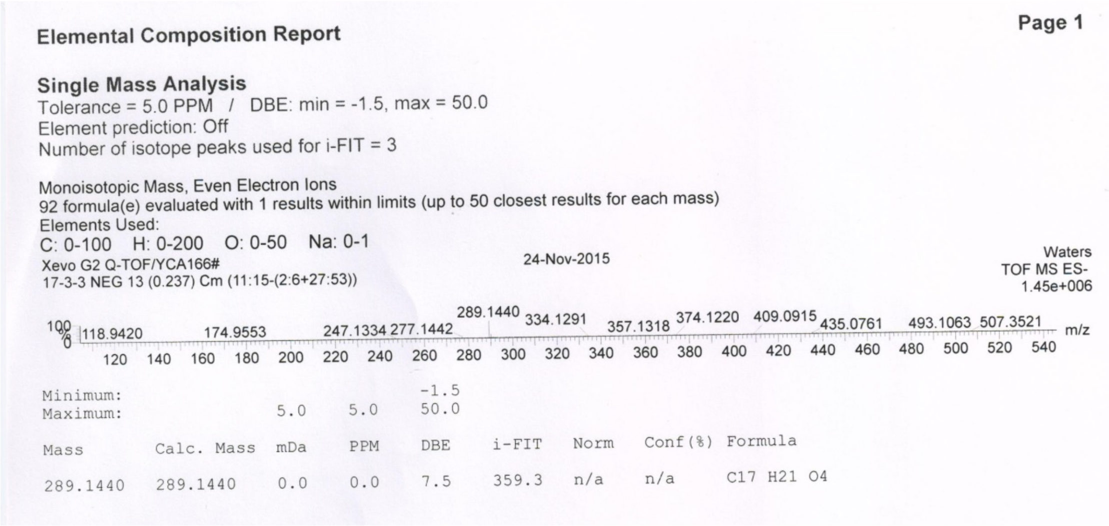
Elemental Composition Report

Page 1

Single Mass Analysis
Tolerance = 5.0 PPM / DBE: min = -1.5, max = 50.0
Element prediction: Off
Number of isotope peaks used for i-FIT = 3

Monoisotopic Mass, Even Electron Ions
92 formula(e) evaluated with 1 results within limits (up to 50 closest results for each mass)
Elements Used:
C: 0-100 H: 0-200 O: 0-50 Na: 0-1
Xevo G2 Q-TOF/YCA166# 24-Nov-2015
17-3-3 NEG 13 (0.237) Cm (11:15-(2:6+27:53))

Waters
TOF MS ES-
1.45e+006



| | Mass | Calc. Mass | mDa | PPM | DBE | i-FIT | Norm | Conf(%) | Formula |
|---|---|---|---|---|---|---|---|---|---|
| Minimum: | | | | | -1.5 | | | | |
| Maximum: | | | 5.0 | 5.0 | 50.0 | | | | |
| | 289.1440 | 289.1440 | 0.0 | 0.0 | 7.5 | 359.3 | n/a | n/a | C17 H21 O4 |

**Figure S3**. HR–ESI–MS spectrum of **1**.

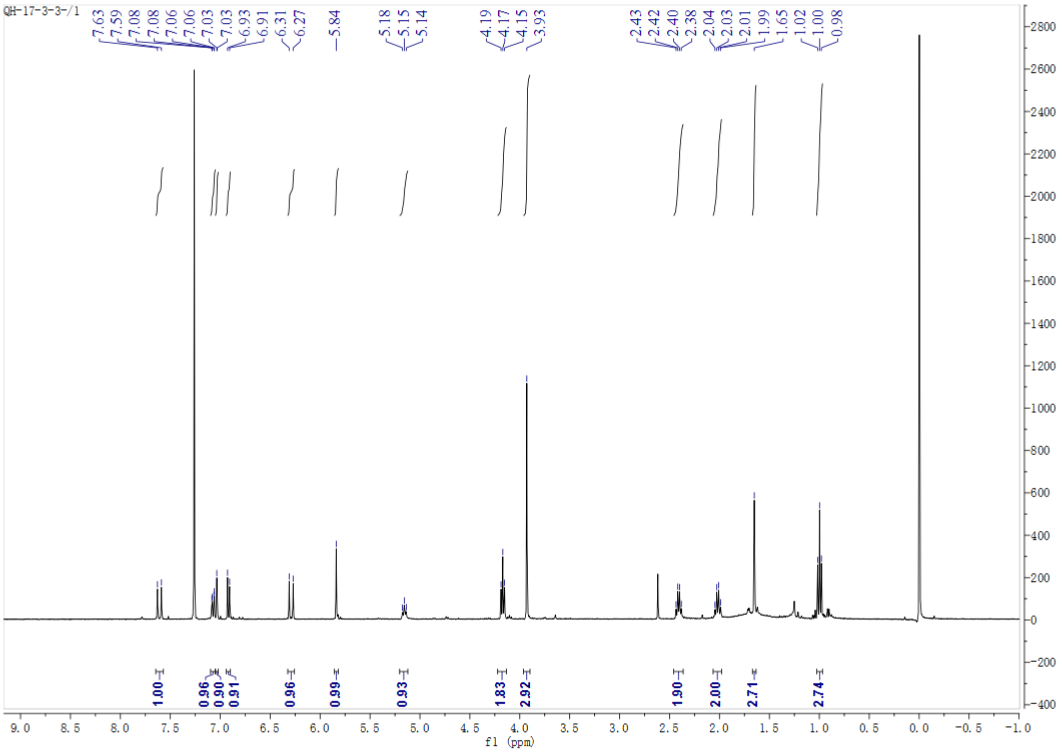


**Figure S4**. $^{1}$H NMR spectrum of **1** in $CDCl_3$.

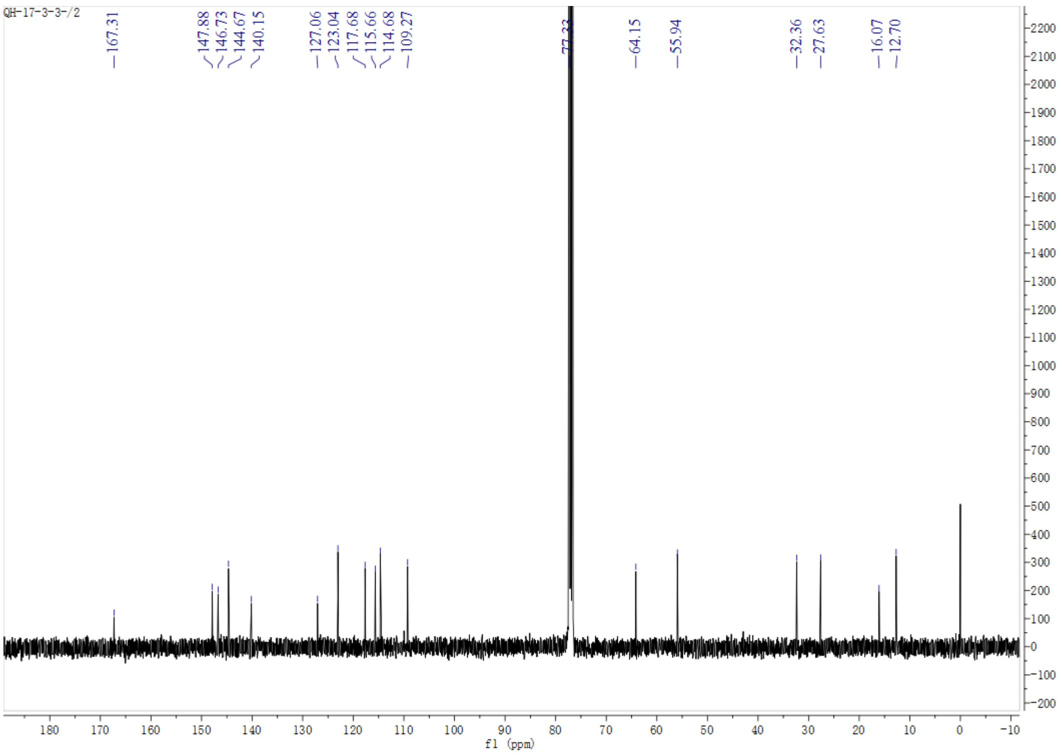


**Figure S5**. $^{13}$C NMR spectrum of **1** in $CDCl_3$.

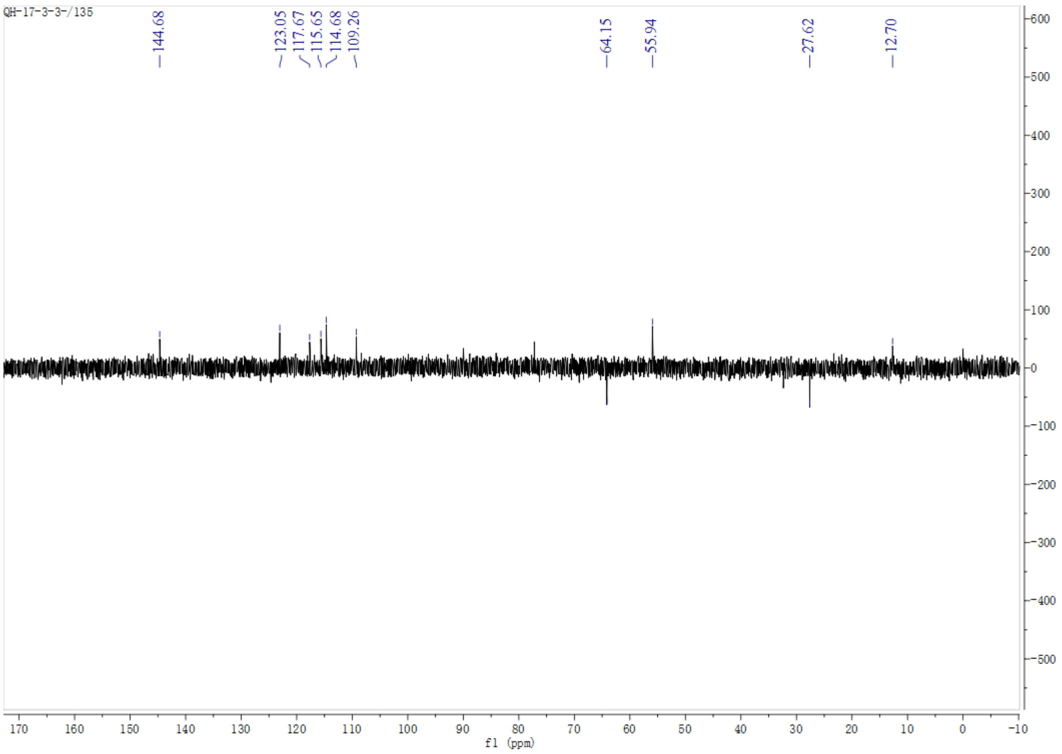


**Figure S6**. DEPT 135 spectrum of **1** $CDCl_3$.

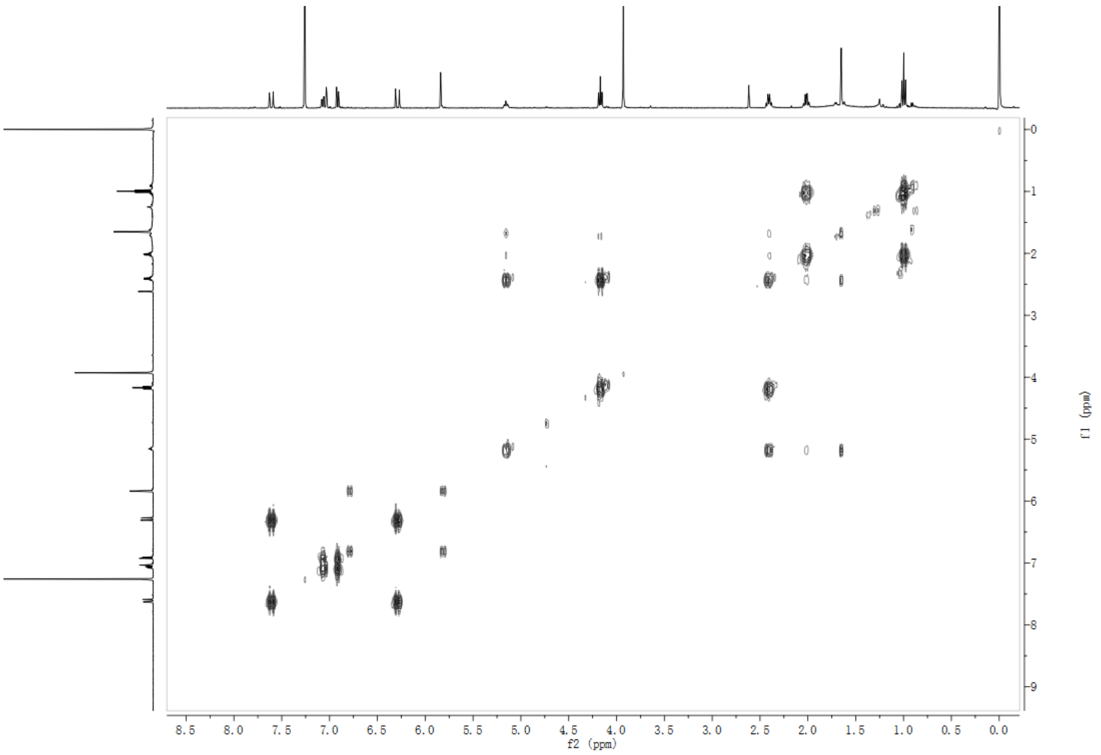


**Figure S7**. $^{1}H–^{1}H$ COSY spectrum of **1** $CDCl_3$.

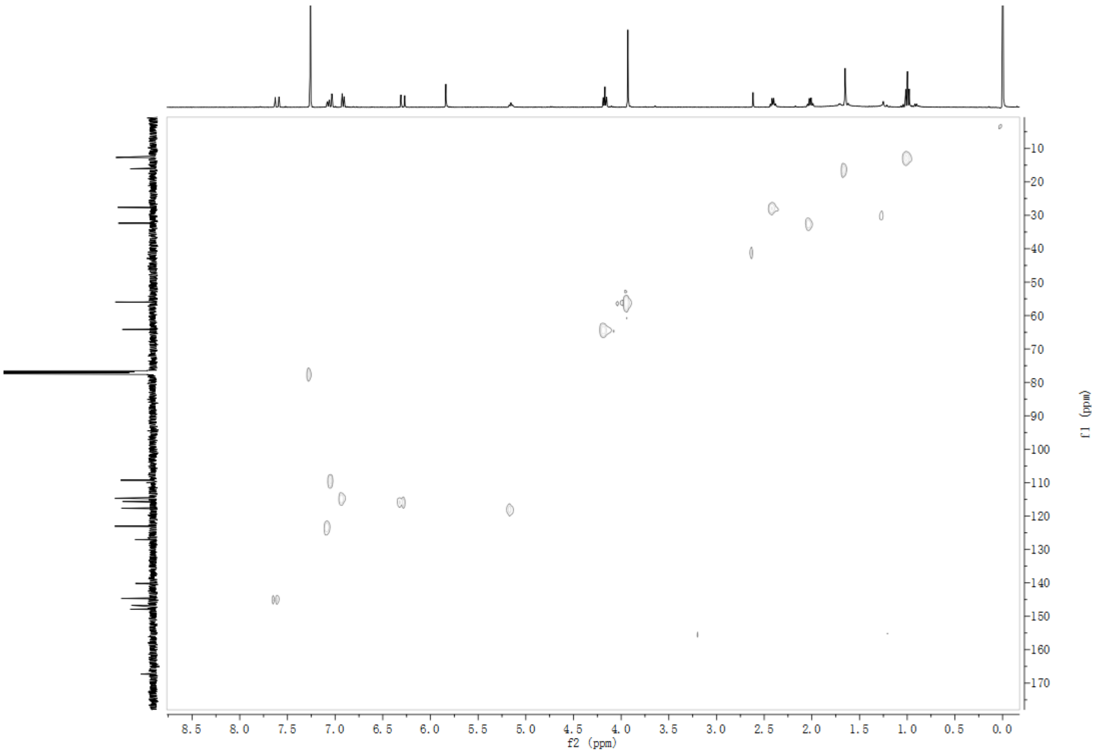


**Figure S8**. HSQC spectrum of **1** $CDCl_3$.

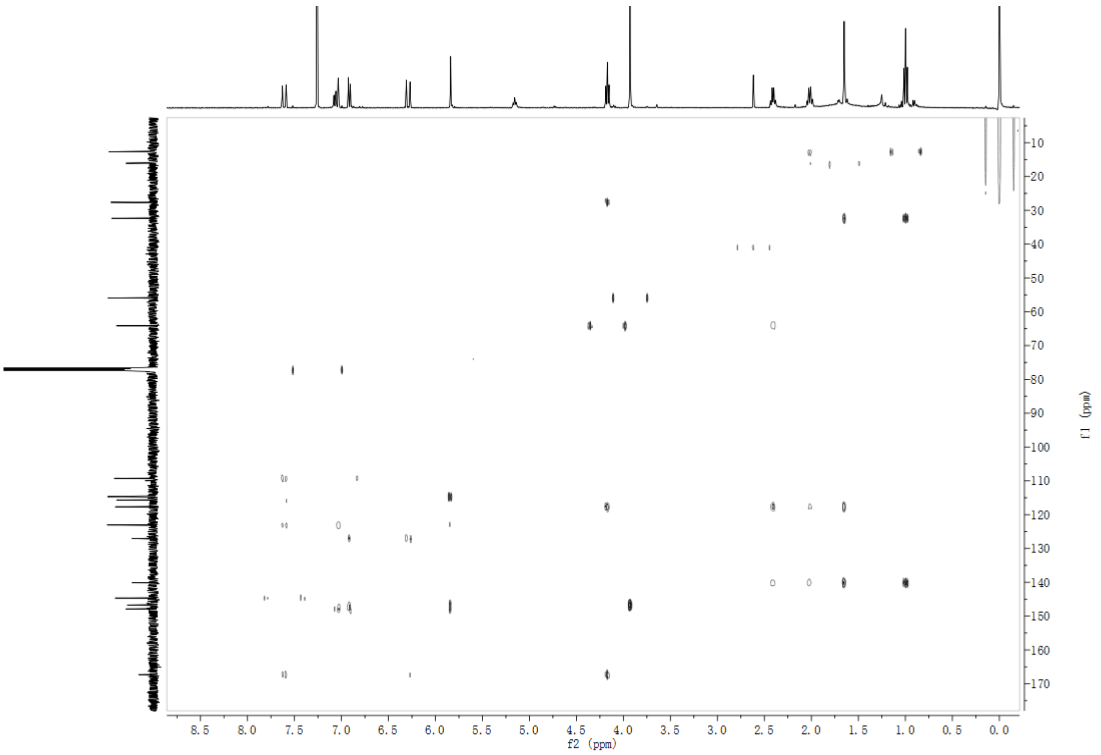


**Figure S9**. HMBC spectrum of **1** $CDCl_3$.

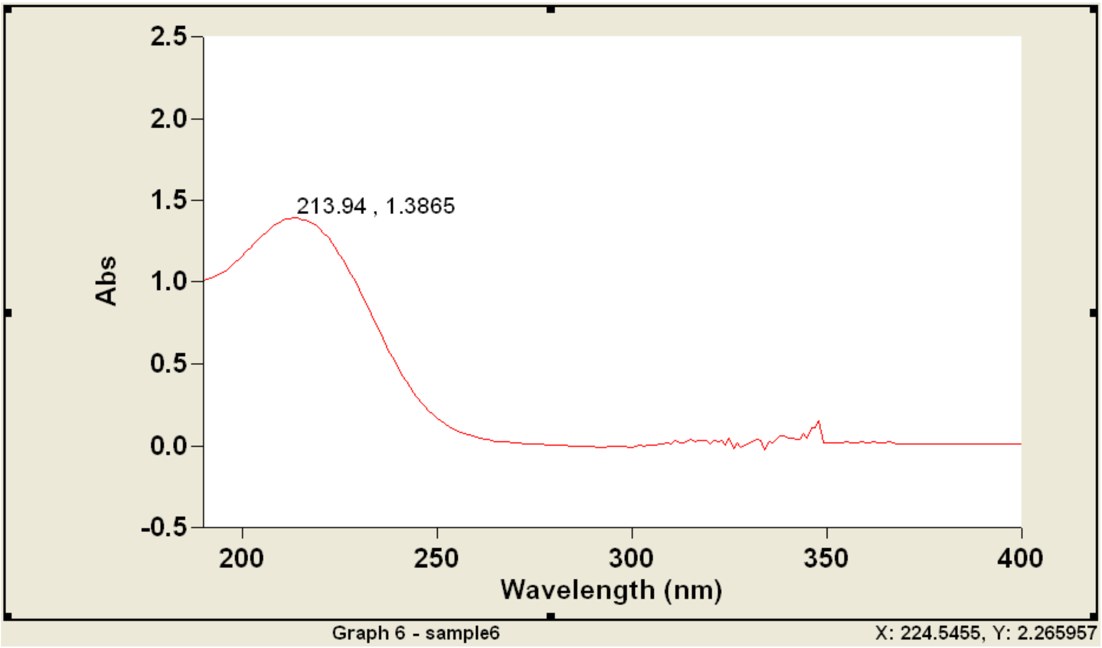


**Figure S10**. UV spectrum of **8** in MeOH.

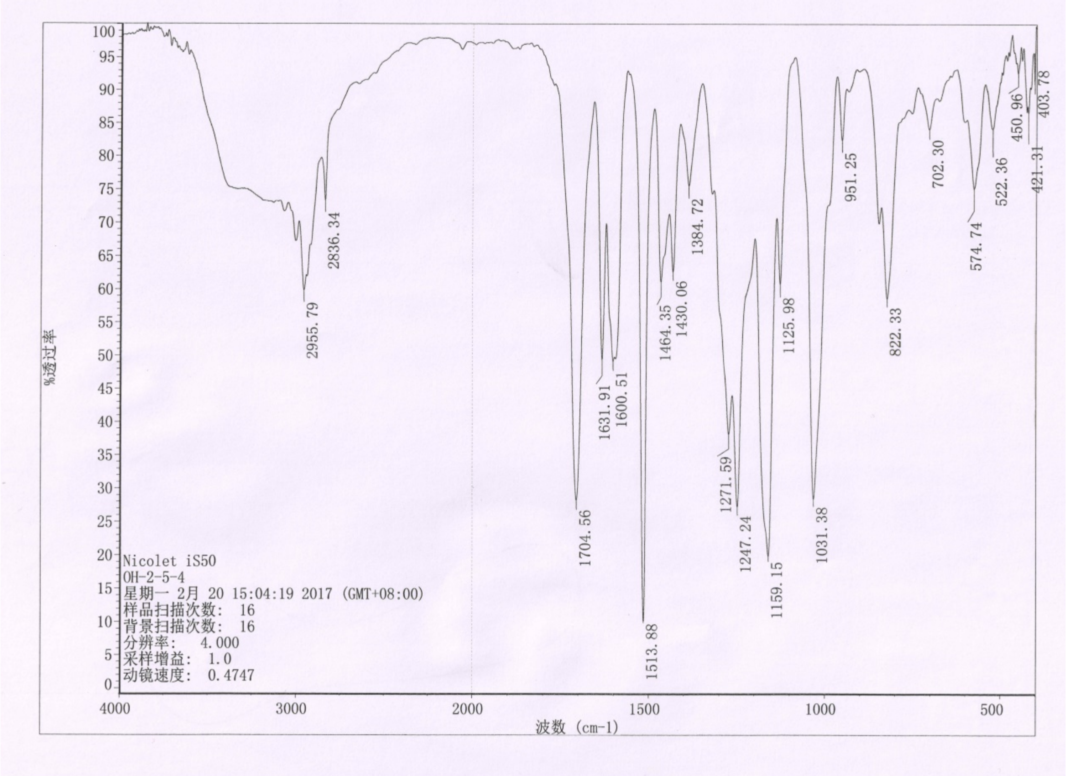


**Figure S11**. IR spectrum of **8**.

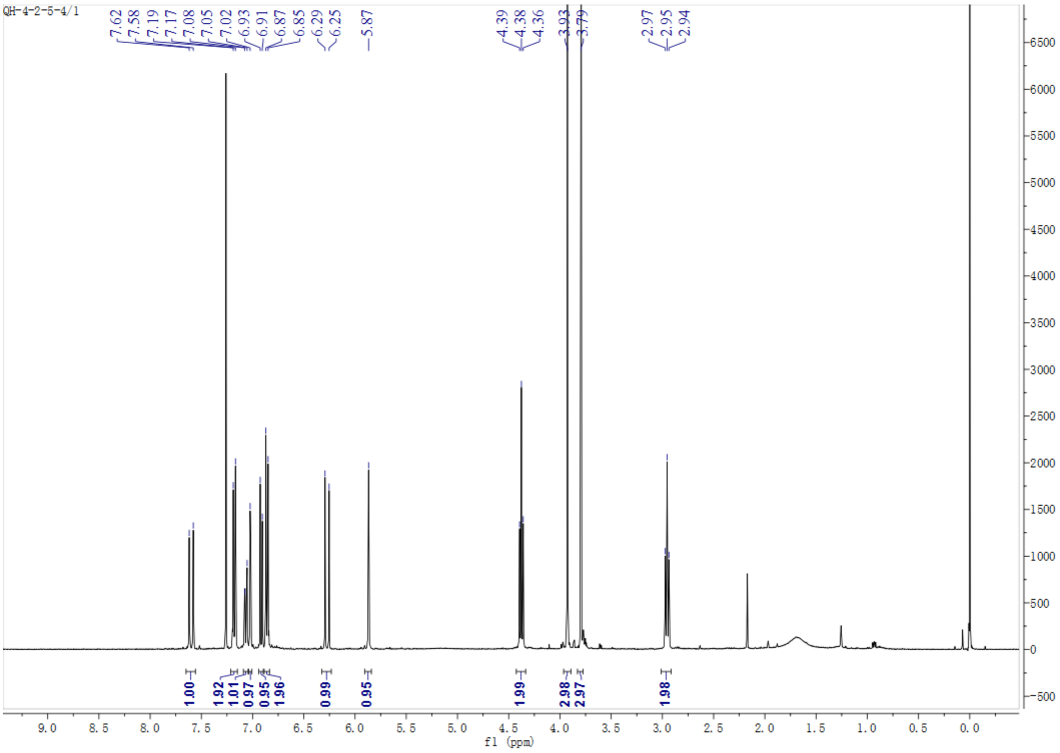


**Figure S12**. $^{1}$H NMR spectrum of **8** in $CDCl_3$.

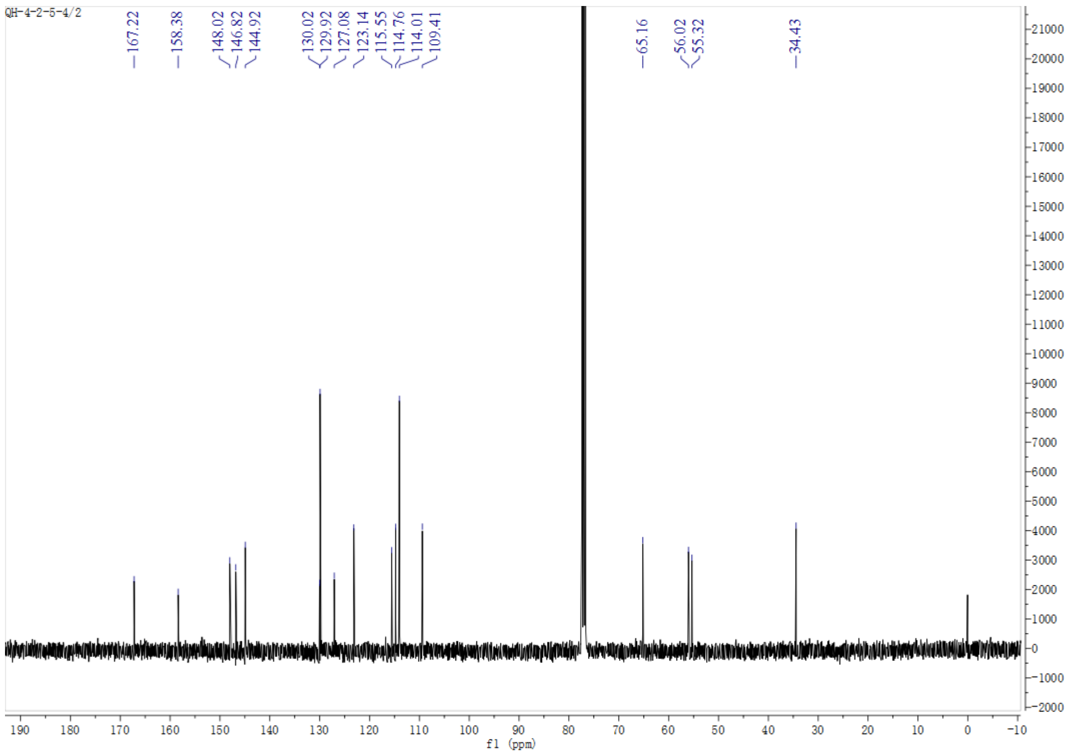


**Figure S13**. $^{13}$C NMR spectrum of **8** in $CDCl_3$.

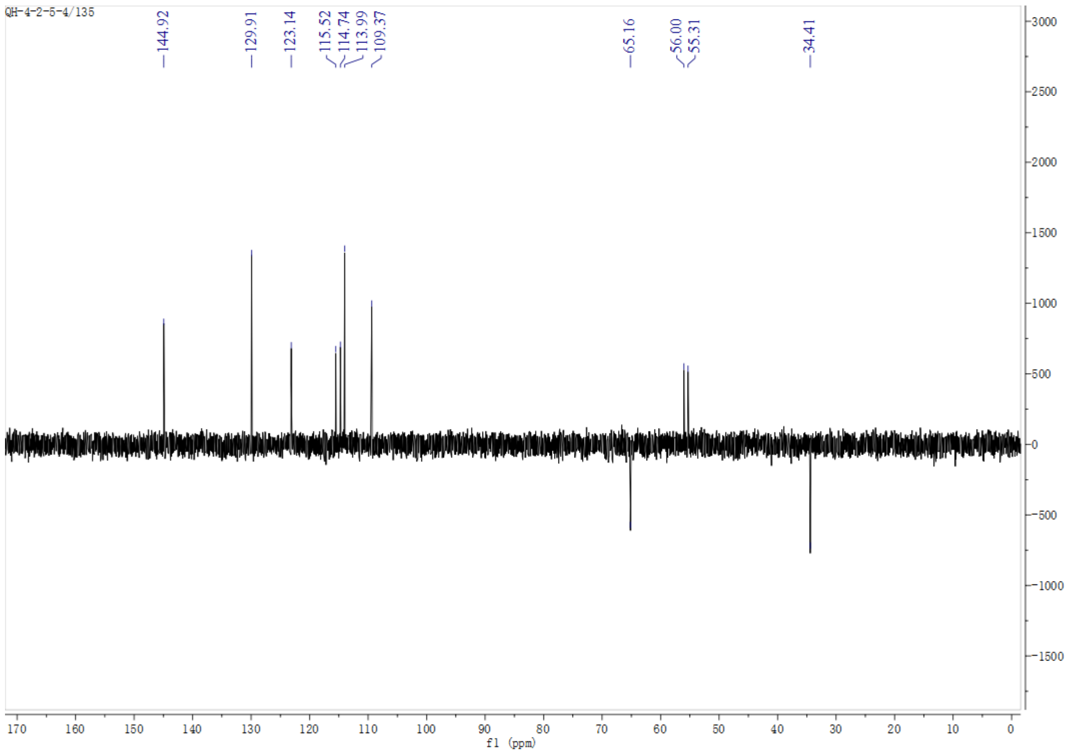


**Figure S14**. DEPT 135 spectrum of **8** in $CDCl_3$.

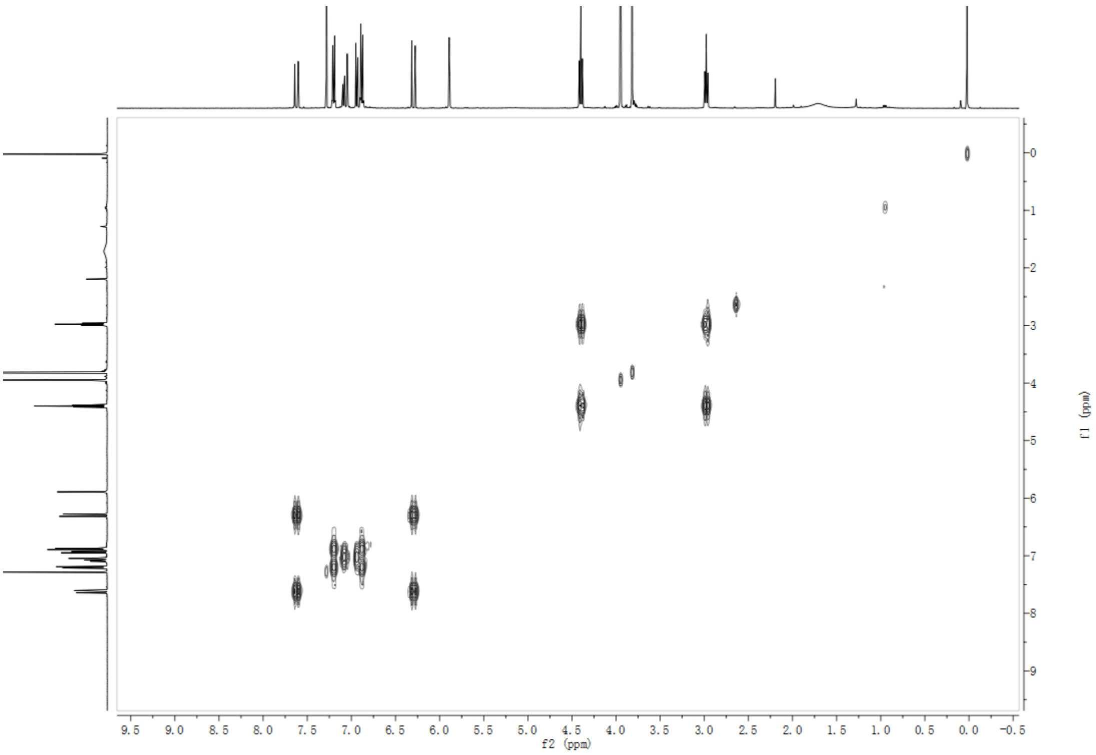


**Figure S15**. $^{1}H$–$^{1}H$ COSY spectrum of **8** in $CDCl_3$.

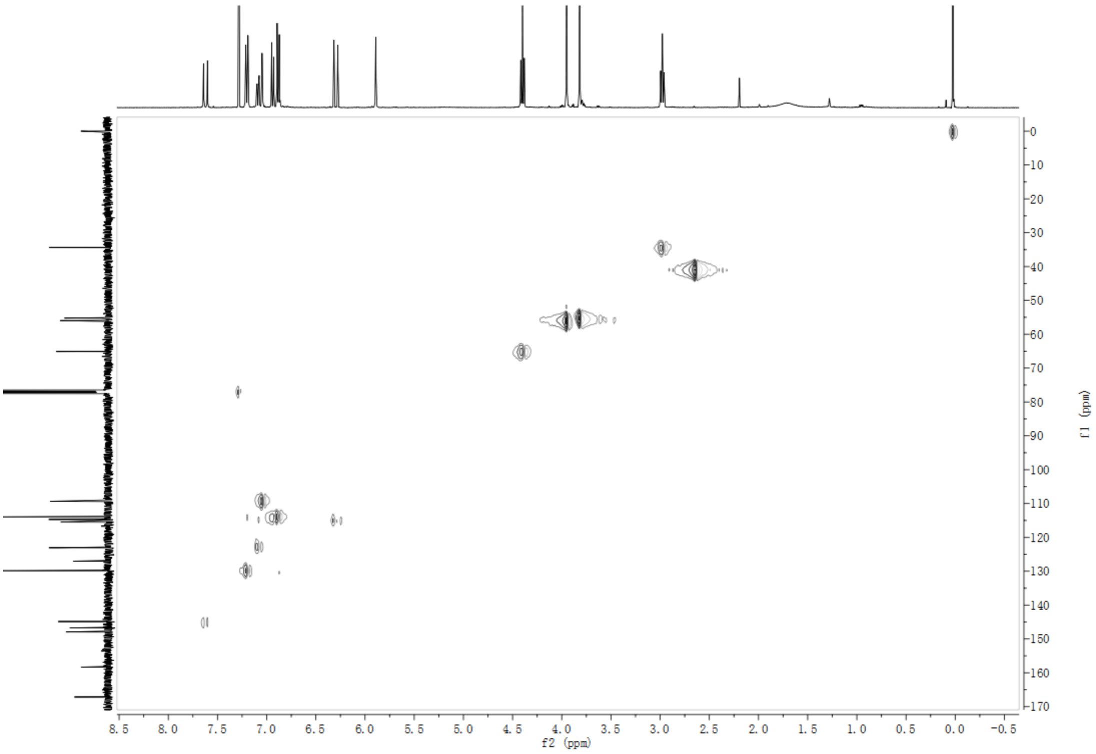


**Figure S16**. HSQC spectrum of **8** in $CDCl_3$.

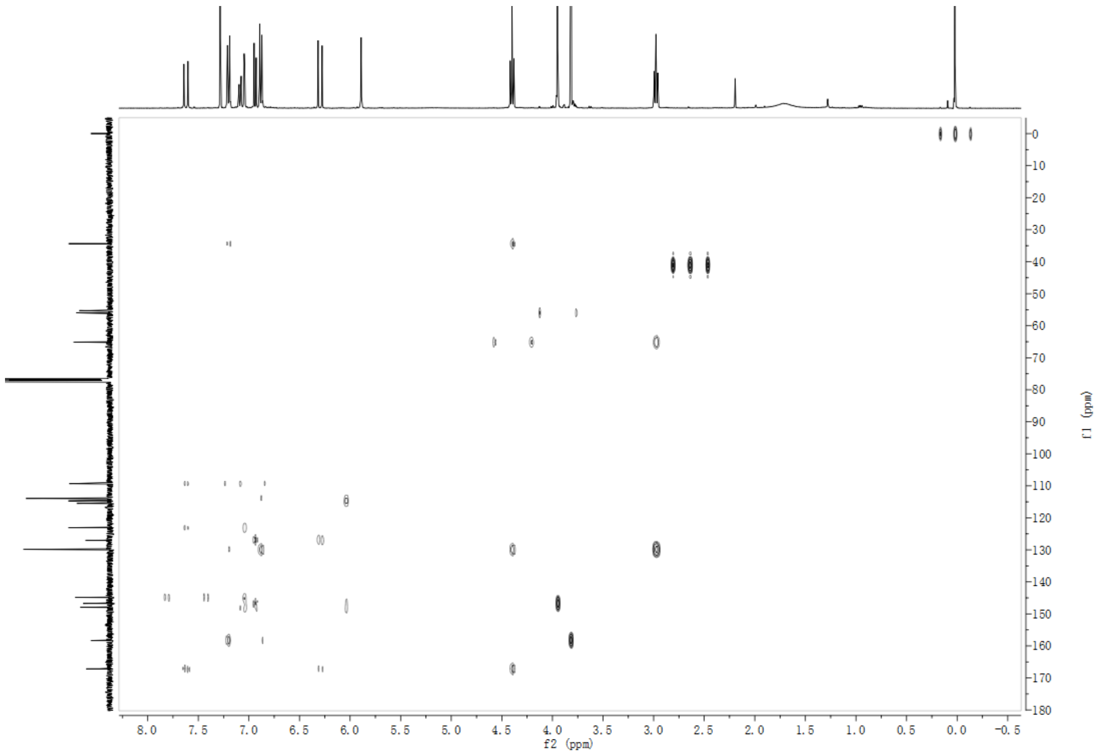


**Figure S17**. HMBC spectrum of **8** in $CDCl_3$.

**Table S1**. Precision, accuracy, recovery, and stability of the phenolic acid esters.

| No. | QC/μM | Precision RSD(%) | | Accuracy (%) | | Recovery (%) | Stability (%) |
|---|---|---|---|---|---|---|---|
| | | Intra-day | Inter-day | Intra-day | Inter-day | | |
| **1** | 5 | 1.98 | 2.88 | 89.54 | 95.72 | 85.13±2.45 | 93.56±1.02 |
| | 25 | 0.63 | 1.54 | 82.13 | 96.86 | 86.66±1.33 | 95.65±0.56 |
| | 50 | 1.23 | 1.36 | 86.04 | 96.23 | 86.25±1.17 | 92.25±1.63 |
| **2** | 5 | 2.30 | 1.99 | 89.65 | 92.58 | 85.56±1.70 | 98.84±2.09 |
| | 25 | 1.26 | 2.33 | 96.98 | 101.59 | 80.50±1.87 | 94.57±1.85 |
| | 50 | 1.69 | 2.01 | 96.22 | 93.25 | 83.39±1.68 | 97.25±2.09 |
| **4** | 5 | 2.03 | 2.35 | 104.24 | 92.27 | 82.28±1.93 | 92.28±2.07 |
| | 25 | 2.20 | 2.14 | 105.16 | 104.15 | 90.41±194 | 100.74±2.15 |
| | 50 | 1.28 | 1.35 | 109.74 | 97.14 | 91.97±1.24 | 95.85±2.01 |
| **5** | 5 | 2.51 | 1.74 | 89.74 | 93.72 | 85.72±1.49 | 96.29±0.99 |
| | 25 | 1.08 | 2.58 | 82.58 | 86.69 | 96.68±2.49 | 94.56±0.96 |
| | 50 | 1.54 | 2.27 | 86.27 | 92.35 | 86.23±1.96 | 95.85±1.01 |
| **6** | 5 | 1.02 | 2.75 | 89.75 | 93.56 | 85.56±2.35 | 95.08±1.56 |
| | 25 | 1.32 | 2.35 | 96.35 | 98.50 | 90.50±2.13 | 102.02±1.96 |
| | 50 | 1.55 | 2.14 | 96.14 | 101.39 | 89.25±1.91 | 102.05±1.39 |
| **8** | 5 | 1.47 | 2.86 | 108.87 | 108.07 | 80.18±2.29 | 93.28±2.17 |
| | 25 | 2.01 | 2.51 | 95.80 | 90.41 | 90.14±2.26 | 95.45±1.12 |
| | 50 | 1.69 | 1.97 | 89.74 | 10114 | 101.79±2.00 | 102.58±1.54 |
| **9** | 5 | 1.37 | 3.27 | 86.60 | 102.09 | 92.45±3.02 | 92.26±2.89 |
| | 25 | 0.98 | 2.91 | 88.43 | 103.08 | 93.50±2.72 | 93.71±1.88 |
| | 50 | 1.03 | 2.04 | 96.06 | 98.92 | 96.91±1.98 | 101.99±1.32 |
